# AI-Based Metamaterial Design

**DOI:** 10.1021/acsami.4c04486

**Published:** 2024-05-29

**Authors:** Ece Tezsezen, Defne Yigci, Abdollah Ahmadpour, Savas Tasoglu

**Affiliations:** † Graduate School of Science and Engineering, Koç University, Istanbul 34450, Türkiye; ‡ School of Medicine, Koç University, Istanbul 34450, Türkiye; § Department of Mechanical Engineering, Koç University Sariyer, Istanbul 34450, Türkiye; ∥ Koç University Translational Medicine Research Center (KUTTAM), Koç University, Istanbul 34450, Türkiye; ⊥ Bogaziçi Institute of Biomedical Engineering, Bogaziçi University, Istanbul 34684, Türkiye; # Koç University Arçelik Research Center for Creative Industries (KUAR), Koç University, Istanbul 34450, Türkiye

**Keywords:** artificial intelligence (AI), metamaterials, biomedical diagnostics, point-of-care, wearable sensors, optics, acoustics

## Abstract

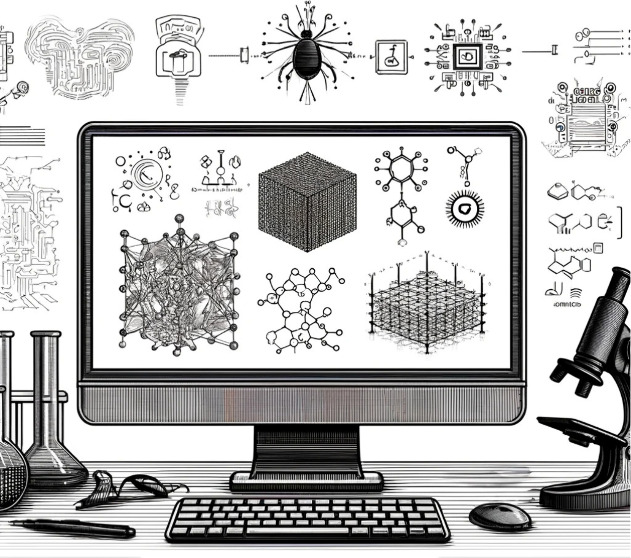

The use of metamaterials in various devices has revolutionized applications in optics, healthcare, acoustics, and power systems. Advancements in these fields demand novel or superior metamaterials that can demonstrate targeted control of electromagnetic, mechanical, and thermal properties of matter. Traditional design systems and methods often require manual manipulations which is time-consuming and resource intensive. The integration of artificial intelligence (AI) in optimizing metamaterial design can be employed to explore variant disciplines and address bottlenecks in design. AI-based metamaterial design can also enable the development of novel metamaterials by optimizing design parameters that cannot be achieved using traditional methods. The application of AI can be leveraged to accelerate the analysis of vast data sets as well as to better utilize limited data sets via generative models. This review covers the transformative impact of AI and AI-based metamaterial design for optics, acoustics, healthcare, and power systems. The current challenges, emerging fields, future directions, and bottlenecks within each domain are discussed.

## 1 Introduction

**Figure 1:**
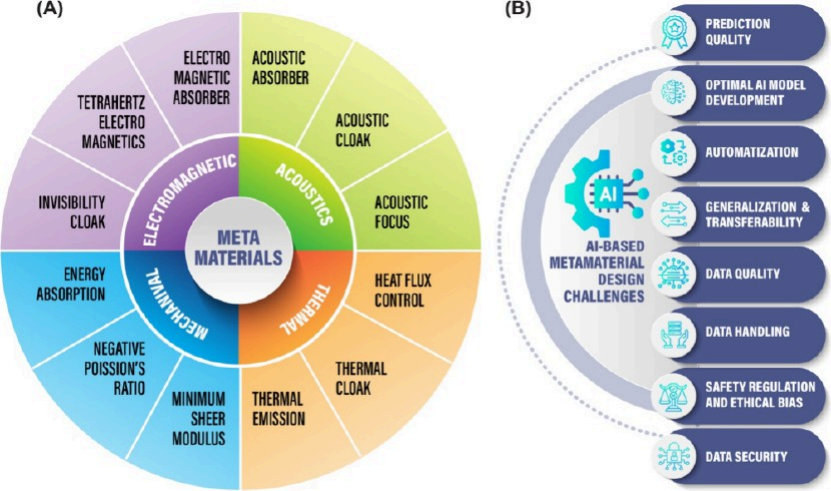
(A) Overview of metamaterial design and applications. The metamaterial technologies are categorized as mechanical, thermal, acoustic, and electromagnetic based on the properties they manipulate and control. (B) Overview of some challenges in AI-based metamaterial design including the quality of AI models in predicting metamaterial function and performance, generation of optimal AI models, achieving automatization, the prospects of generalization and transferability across different metamaterials, data quality, data handling, constructing models regulated for safety, ethical conduct, and bias, and achieving data security. Some elements in this figure were designed using resources from freepik.com.

In a rapidly evolving world, global challenges require innovative and interdisciplinary solutions. The fields of acoustics, optics, healthcare, and power systems emerge as pivotal avenues for innovation and have the potential to drastically improve the quality of human life. Metamaterials have gained attention in recent years due to their potential to revolutionize various fields.^1,2^ They offer unusual properties including unique electromagnetic, thermal, and acoustic (Table 1)^3,4^ and mechanical characteristics (Figure 1A).^5,6^ They offer a promising solution to overcome the limitations of conventional materials.^7−9^ Moreover, with the aging population and rising medical demands, healthcare can greatly benefit from AI-driven solutions.^10^ WHO expects to present an upcoming global scarcity of healthcare excess by 2030.^11^ AI-based designs can alleviate the workload of data interpretation or novel technology development in the fields of medical imaging,^12^ drug delivery,^13,14^ and personalized medicine,^15−17^ thus enhancing healthcare accessibility, affordability, and efficacy.^18^ Optics can enable breakthroughs in computational power such as quantum computers,^19^ communication,^20^ solar power generation,^21^ and medical diagnostics,^22^ paving the way for imagining systems,^22^ information,^23^ and data analysis.^19^ The current crisis of energy security,^24^ environmental conservation,^25^ and industrial resilience,^26^ can undergo transformative shifts driven by AI-enabled metamaterials and smart grid technologies. Power transfer and harvesting ushers decentralized energy production,^27^ grid optimization,^28^ and renewable energy integration,^29^ thereby addressing energy challenges while fostering economic growth and environmental sustainability. Acoustics plays a crucial role in communication,^30^ medical treatments^31^ and environmental sustainability.^32^ By harnessing these four fields and their intercourses, researchers can tackle multifaceted challenges and demands of a growing population and limited resources toward a better world.

**Table 1:** Summary of Metamaterials Domains

Domain	Applications	Advantages	Limitations	AI models	Ref
Acoustics	Absorber	Tunability	Frequency-dependent	Evolutionary	(43)
	Cloaking	Real-time adjustment	Durability	Algorithms	
	Lens	Total absorption	Fatigue	Swarm intelligence	
		Broadband	Manufacturability	Algorithms.	
Optic	Meta-lens	Chromatic dispersion	Scalability	Deep neural network	(44−47)
	Meta-grating	High-resolution imaging	Adaptability	Neural network	
			Miniaturization	Conventional neural network	
Healthcare	Diagnostics	Biomimetic	Sensitivity	Finite element model	(1,48,49)
	Point-of-care	Non-invasive	Liability issues	Extreme randomized tree	
	Auxetic	Non-destructive	Compliance issues		
		Wave-front modulation	Power consumption		
			Biocompatibility		
Power	Power harvesting	Low power	High-cost	K-nearest neighbors (KNN)	(50,51)
	Power Transfer	Consumptions	Scalability	Linear regression	
		Customization		Decision trees	
		Safety		Neural networks	

The convergence of artificial intelligence (AI) and metamaterials also offers new design possibilities.^33^ Traditional metamaterial design methods often rely on labor-intensive trial-and-error approaches.^34^ This limits the exploration of the vast design space and results in lower efficiency outcomes. In contrast, AI-based procedures leverage machine learning (ML) algorithms to automate and accelerate the design process, facilitating the discovery of novel metamaterial structures with enhanced performance.^35^ The frameworks usually involve initial training in which computational models learn the desired properties of the metamaterial and its structural parameters, thus generating new designs or optimizing existing ones to achieve specific objectives.^36,37^ Regression and probabilistic algorithms evaluate complex structures and combinations of unit materials, while probability algorithms are primarily utilized for designing novel metamaterials.^38,39^ The literature contains three subcategories of AI usage in metamaterial design: data analysis, algorithm development, and model evaluation.^1,40^ Before any AI-based application, data sets undergo data preprocessing and assessment. Raw data in metamaterials are often harvested from experimental or numerical simulations and can be incomplete or inconsistent, or contain background noise.^41^ Data preprocessing such as cleaning is commonly used to rearrange and compose relevant sections of data. Incomplete and noisy data can be cleaned via ML.^42^

AI models used to design metamaterials can be categorized as supervised, unsupervised, or hybrid models.^52^ A general subdivision can be generative models and optimization algorithms. Generative Models such as generative adversarial networks (GANs) and variational autoencoders (VAEs)^38^ play a role in the generation of novel metamaterial designs by exploring latent spaces and patterns which would be labor intensive using traditional manual methods. In general, GANs can create structures and architectures, while VAEs can present novel metamaterial configurations with tuned and desired functionality.^53^ Optimization algorithms such as genetic algorithms and particle swarm optimization explore the design space of metamaterials.^54^ Genetic algorithms use the principle of natural selection and particle swarm optimization operates with the principle of adaptation which can be utilized for fine-tuning metamaterial architecture.^55^ Cross-comparisons of models are summarized in Table 2.

**Table 2:** Machine Learning and Artificial Intelligence Types with Their Advantage and Disadvantages^a^

Type	Method	Advantages	Limitations	Ref
Single method	SVM	Structural risk minimization	Parameter uncertainty	(57)
	GP	Optimal search	Lack of memory	(58)
	PSO	Memory storage	Local minimization drawback	(59)
	FEM	Complex problems	High cost	(60)
Hybrid method	RF	Data characterization	Slow	(61)
	ANN	Input selection	Overfitting	(62)
	DNN	Fast searching algorithm	Complicated architecture	(63)
	GAN	High efficiency	Data hungry	(64)

a Fl: federated learning, GP: Gaussian process, PSO: particle swarm optimization, FEM: finite element analysis, RF: random forest, ANN-K shape: artificial neural network K shape, ANN: artificial neural network, DNN: deep neural network, GAN: generative adversarial networks (GANs). Reproduced with permission from^56^. Copyright 2018 MPDI.

In terms of metamaterial design, generative models and optimization algorithms stand out.^65^ Generative models excel in creativity, producing novel concepts, whereas optimization algorithms focus on efficiency and convergence to optimal solutions. Lastly, model evaluation assesses the performance of models by cross-comparison and prediction quality. Models should be able to perform accurate predictions while avoiding overfitting.^66^ The assessment of the models is performed via performance indices, bootstrapping, and cross-validation.^1,67,68^ By utilizing AI, researchers can efficiently explore complex parameter spaces, enabling the discovery of metamaterials.^69^ Several questions regarding the data availability to train models remain to be addressed for design accuracy. We will dive deep into the next section and present emerging applications in acoustics, biomedical engineering, optics/photonics, power harvesting, and others.

## 2 Emerging Applications of AI-Based Design in Acoustic Metamaterial

The discipline of acoustic metamaterials, which involves the engineering of materials to control acoustic properties, is undergoing a transition with the integration of AI and ML. Traditionally, the design of acoustic metamaterials has depended on time-consuming trial-and-error techniques. Design accuracy is an important factor for narrow cavities where thermoviscous loss must be considered.^70^ AI-based design methodologies are transforming the field via computational models and ML algorithms. Moreover, such design strategies can uncover patterns, optimize designs, predict the acoustic performance of new materials, and analyze different variations. In recent years several groups have explored the use of AI-based design for sound cancellation, noise control, lenses, cloaking absorbers, and acoustic sensors.^2,71,72^

### 2.1 Acoustic Absorbers

The design of low-frequency noise reduction or local insulation is a challenging field of noise control. The procedure requires a specific sound insulation frequency and higher-level optimization in local attenuation.^73^ In recent years some groups have utilized ML and neural networks (NNs) to accelerate the design and iteration evolution of metamaterials while keeping the design requirement of thickness, local attenuation, and structure-bone noise blockage.^23,24,38^

Achieving local enhancement through sound field regulation is critical in acoustics research. The degree of attenuation is typically determined based on actual engineering that directs all iterations designed by ML to have the designated working conditions. In the presence of coherently coupled weak resonances (CCWRs), it is difficult to achieve optimal broadband sound absorption. Chen et al. have presented an ML-assisted subwavelength sound absorber with CCWRs using an improved Gauss–Bayesian model.^39^ The initial structure was proposed as an aperiodic structure that comprises three parallel split-ring units with quasi-symmetric resonant mode. Within 80 iterations, the model had determined the optimal CCWRs with sound absorption spectrum (α > 0.9) from 229 to 457 Hz and a broad bandwidth of 44.8%. The framework also decreased the restriction of analytical models and complex aperiodic components by combining AI and the optimal design of metamaterials.^74^ Alternatively, Peng et al. presented membrane-type acoustic metamaterials (MAMs) for insulation via coupling the finite element method (FEM) and Kriging surrogate model. Influences of both material and structural parameters of the membrane and mass block were investigated by first using a single variable control method and the coupling effect. During multiparameter coupling, the influence law of a single variable on the sound insulation peak of MAMs was unchanged. The Kriging surrogate model could design acoustic metamaterials with a specific sound insulation frequency and bandwidth.^75^ A similar approach to FEM in acoustic design was utilized by Zhao et al.^76^ The AI-based design presented a great advantage for sound insulation of a specific frequency range. However, several questions regarding the half-life and durability of AI-based design remain.

Currently, most acoustic absorbers have been designed for ultrathin sizes to achieve perfect and low-frequency sound absorption or insulation performance. This prioritization often results in a compromise of fatigue damage in practical engineering applications.^77^ In membrane-type acoustic metamaterials, the sound waves may cause fatigue damage to the membrane and thus a loss of functionality. To address this, AI can be used to design fatigue-tolerant metamaterials for acoustic absorbers while maintaining absorption performance. One group was able to significantly alleviate fatigue damage via a proposed lightweight optimization for MAMs.^78^ To maintain the low-frequency sound absorption performance while keeping the lightweight purpose, a multiobjective particle swarm optimization algorithm was applied. The fatigue simulation results showed that the corresponding minimum fatigue life was increased by 10.27% using the proposed lightweight approach.^78^ Another example of fatigue can be caused by structure-borne noises. In acoustics, the propagation of elastic flexural waves in plate and shell structures is a common transmission path of structure-borne noises.^55,66^ The noises can result from an impact or a vibration of the adjacent surface and can affect the sound fluctuations in the system. The designs with a frequency band gap can effectively block elastic waves in a certain frequency range. Liu et al. have demonstrated a deep learning (DL)--based workflow for acoustic absorbers. The frameworks showed that, within 360 sets of data for training and testing, the NN attained a 2% error in achieving the target band gap via five design parameters for flexural waves around 3 kHz.^24^ Several authors have recognized the fatigue and its effects. The mentioned example shows that AI can partially if not completely resolve the issues of fatigue and half-life of acoustic metamaterial.

**Figure 2:**
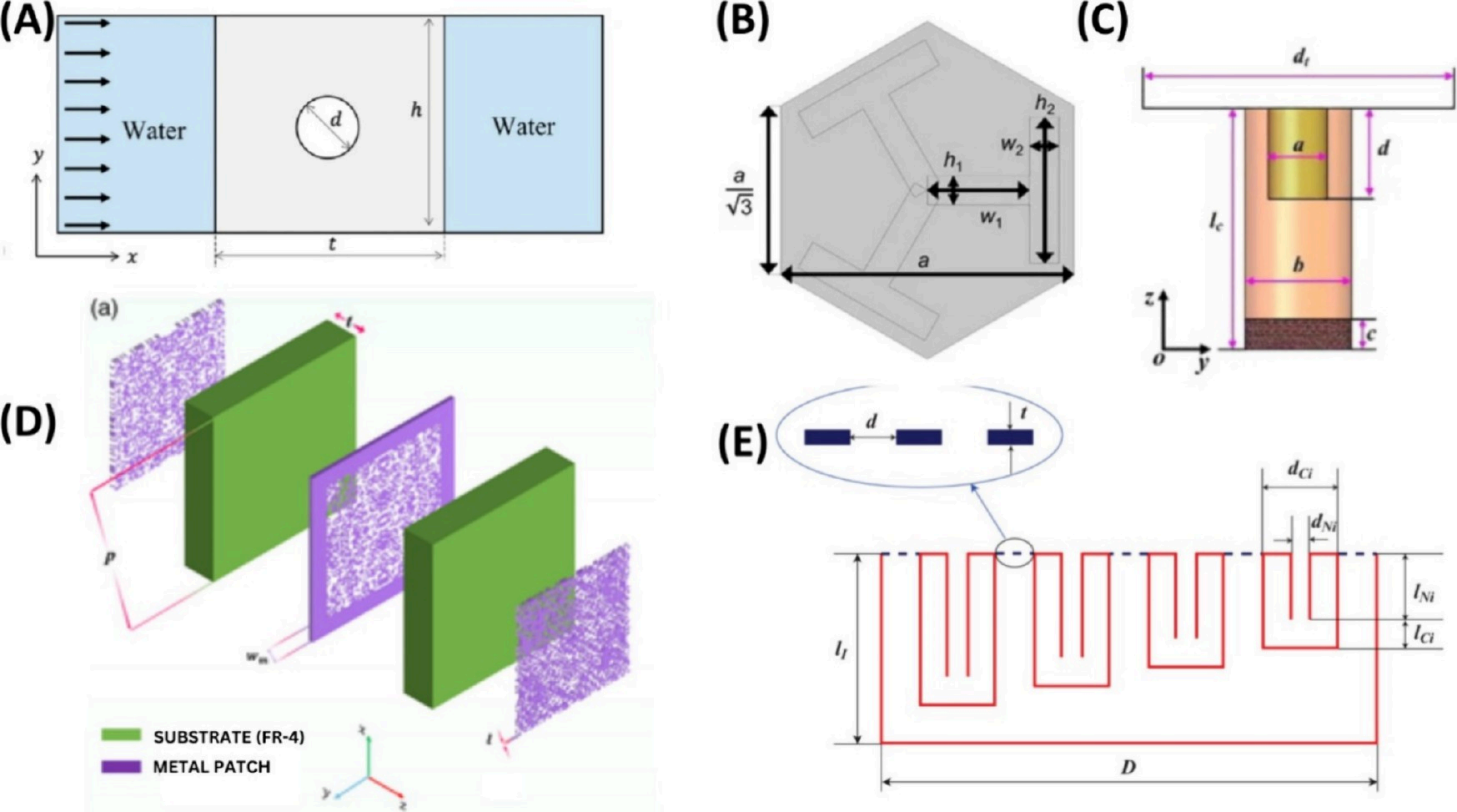
Acoustic absorbers and AI-based design (A) Underwater acoustic absorbers with polyurethane (PU) acoustic coatings. Adapted from ref (80) in accordance with its CC BY 4.0 license. (B) DL-based acoustic metamaterial design for attenuating structure-borne noise in auditory frequency bands. Adapted from ref (300) in accordance with its CC BY 4.0 license. (C) ML inversion design and application verification of a broadband acoustic filtering structure. Adapted with permission from ref (86). Copyright 2022 Elsevier. (D) Microwave metasurface. Adapted from ref (83) in accordance with its CC BY 4.0 license. (E) Design of a Helmholtz resonator (HR) via DNN-based inverse prediction mechanism. Adapted with permission from ref (85). Copyright 2021 AIP Publishing.

The Fano resonance is a widespread wave-scattering phenomenon associated with an ultrasharp line shape, which serves a narrow working frequency range around the interference frequency, rendering the realization of the Fano-based application extremely challenging. Xu et al. have employed an inverse design using Bayesian ML to search for the optimal broadband insulating performance with a rapid convergence speed of 15 iterations.^79^ The design consisted of a symmetric profile and tunable low-frequency that could function as a double-helix metal silencer. The group demonstrated the effectiveness of the proposed silencer with tunable sound attenuation (>90%) in 425–865 Hz and high ventilation (>80%) at various double-helix combinations. The inverse design via rapid convergence speed of lower iterations was able to lower the computational costs. An important question associated with acceleration is its effect on the manufacturability of design and real-life applications. The acceleration of inverse design can have industrial applications and feasibility by minimizing production bottlenecks.^2^ Some groups have combined ML algorithms with NNs to have an accelerated design timeline. Accelerated inverse design of acoustic coating was proposed by Weeratunge et al. for underwater acoustic absorbers.^80^ Polyurethane (PU) acoustic coating (Figure 2A) with embedded cylindrical voids was designed by considering the frequency-dependent viscoelasticity of PU. The viscoelasticity was used instead of a constant frequency-independent complex modulus in the matrix which does not reflect real-life acoustics. The FEM was replaced with an efficiently computable surrogate model developed through a deep neural network (DNN) and demonstrated an increased speed of predicting the absorption coefficient by a factor of 4.5 × 10^3^ and with an accelerated timeline.

Donda et al. have utilized an AI-based acoustic metasurface absorber modeling approach to reduce the characterization time via a conditional generative adversarial network (CGAN). They proposed broadband low-frequency absorbers with coupling unit cells. The method outperformed conventional frameworks and within 5–30 s was able to generate optimal structure with tailored acoustic absorption peaks. The group also developed an ultrathin metasurface absorber that has absorption at an extremely low frequency of 38.6 Hz with an ultrathin thickness down to λ/684 (1.3 cm).^81^ Similarly, Gurbuz et al. developed a method to design acoustic metamaterials based on CGAN.^82^ The specific network type allowed the user to condition with class labels, enabling additional training to differentiate variant labels. The framework used CGAN to find the underlying relation between transmission loss and cell geometries and achieved the inverse design of structural unit cells for the desired sound insulation purpose. The framework had some limitations due to the synthetic data set in training data and its enrichment. Some of these limitations could be overcome by concentrating on the composition of the training data to enrich the GAN with enhanced geometries to broaden the spectrum of possible designs. Finer frequency step sizes and viscothermal effects could be considered to mimic likely resonance behavior. Kiani et al. also presented a similar approach for a microwave metasurface although it was not replicated by other groups, possibly due to the limitations of data (Figure 2D).^83^ The CGAN studies are data-hungry, making them vulnerable to data availability, despite the utilization of synthetic data sets or data enrichment. Due to this, there is a wide choice of convolutional neural network (CNN) models, available in the literature. While CGANs are generative models that can generate new examples from a given training set, CNNs are primarily used for classification and recognition tasks. One group, Zhou et al. developed a CNN-based framework that utilized the relationship between ground pressures at multiple points for larger regional control of sound waves.^84^ The ML version of the framework for designing metasurface had higher accuracy than the genetic algorithm. Some groups including Mahesh et al.^85^ and Cheng et al.^86^ have developed a DNN-based inverse prediction mechanism to geometrically design a Helmholtz resonator (HR) acoustic absorber for low-frequency absorption. By deploying a DNN and autoencoder-like network, both groups observed a predicted center frequency deviation of 9–12 Hz, a relatively acceptable design requirement (Figure 2E). Lai et al.^87^ combined Wasserstein generative adversarial networks with CNN to reduce scattering on metamaterial surfaces. A similar CNN approach was also utilized in the CNN-Genetic algorithm (GA) to accelerate the iteration timeline and evolution.^81,88^

A considerable body of literature on cloaking applications exists for acoustic absorbing. A closer look at the literature reveals several gaps. These include environmental sensitivity, complex fabrication, and limited frequency ranges. In addition, scalability and cost-effectiveness are major concerns. AI assistance can help overcome some of these challenges and enable the development of versatile devices. Furthermore, generative algorithms, NNs, and simulations have already addressed some metamaterial design complexities. Multiphysics simulation tools can further assist AI-based tools in exploring a broader design space. AI-integrated generative design techniques have been used to design multifunctional and adaptive/tunable absorbers, broaden frequency ranges, and enable bioinspired designs. Future directions include the development of an AI-driven adaptive absorber that can continuously monitor the environment and dynamically adjust absorption in real time. In the field, such advances can have significant implications for stealth technology and can be used in active combat regions or for security purposes.

### 2.2 Acoustic Cloaks

**Figure 3:**
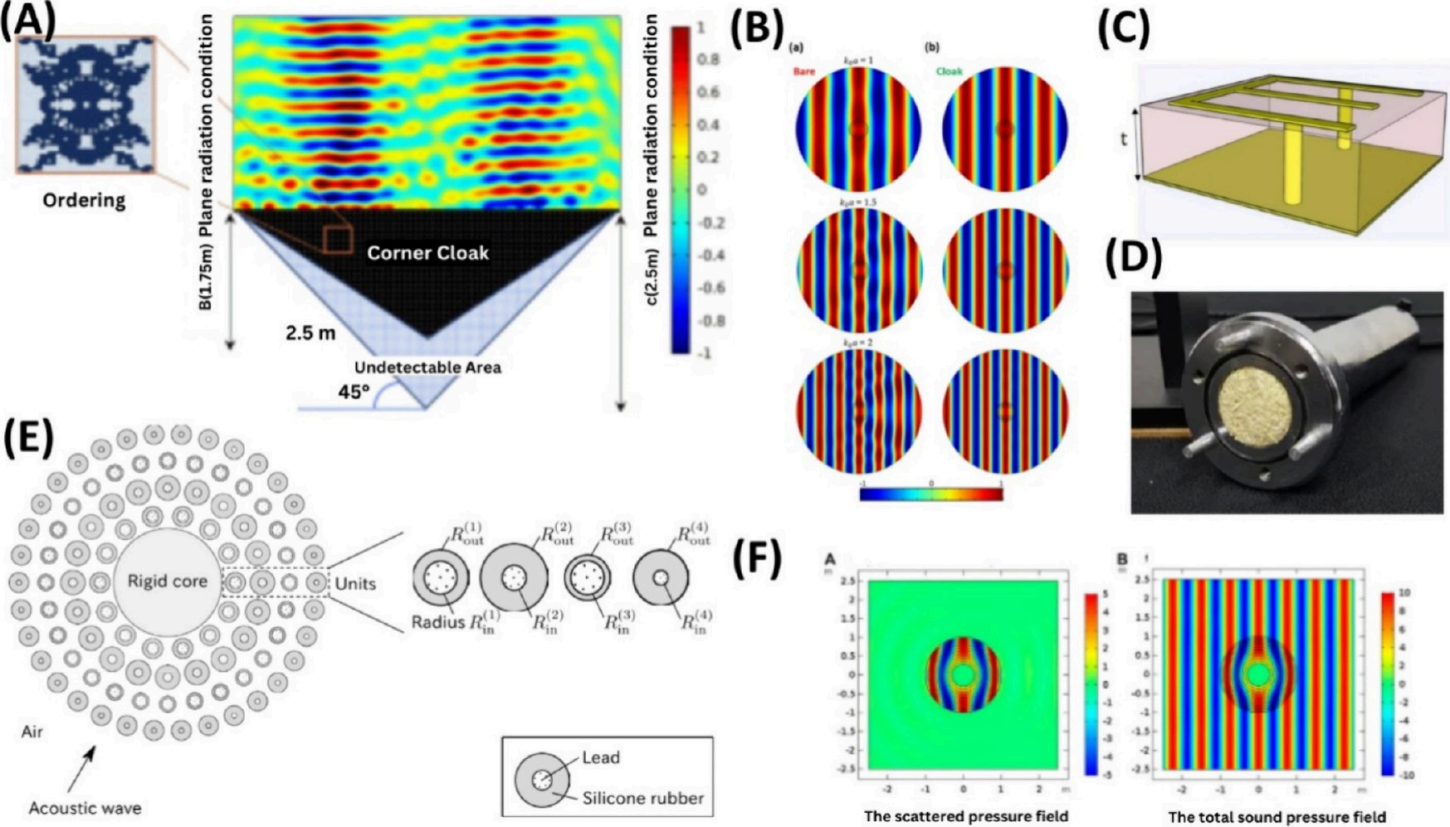
Acoustic cloak and AI-based design. (A) Acoustic corner/cloak via FEM. Adapted with permission from ref (101). Copyright 2021 Elsevier. (B) Machine-learning-driven acoustic invisibility cloak with a multilayered core–shell configuration. Adapted from ref (104) in accordance with its CC BY 4.0 license. (C) Carpet cloaks operate for a wide range of incident angles using a DNN and PSO algorithm. Adapted from ref (301) in accordance with its CC BY 4.0 license. (D) Sugar cane bagasse-based acoustic cloak composite using artificial NN model. Adapted from ref (107) in accordance with its CC BY 4.0 license. (E) Omnidirectional acoustic cloaking against airborne sound is realized by a locally resonant sonic material. Adapted from ref (302) in accordance with the CC BY 4.0 license. (F) Acoustic cloaking design for the underwater environment. Adapted from ref (106) in accordance with its CC BY 4.0 license.

Acoustic cloaking has been recognized as an emerging field of acoustic research.^89^ Variant methods have been proposed to obtain cloaking.^90^ Manipulation of synthetic materials such as metamaterials, phononic crystals, transformation optics, integration of surfaces, and carpet cloaking are examples of some methods.^91−96^ Primarily, acoustic cloaking can be achieved by controlling the sound wave and its flow within the environment and material. The control of a wave requires a material with an anisotropic and spatially distributed structure.^97^ If the sound wave can bypass the object and any scattered sound can be removed, the cloaking phenomenon can be observed. The requirement can be obtained by changing the geometric patterns of a microstructure until the targeted wave control is observed.^98^ In comparison to traditional materials, metamaterials^99^ might be a cheaper option to iterate a spectrum of wave control or tunability.^100,101^ In recent years, some groups have explored ML algorithms to achieve rapid and accurate acoustic cloaking via novel metamaterial designs. Zhang et al. have proposed an inverse design method based on a FEM of ML.^101,102^ The group established a digital structural genome to combine FEM with design production and calculate wave properties of digital metamaterials with multiple iterations and microstructure orientation (Figure 3A). The database contained the anisotropy and spatial distribution of metamaterials. The group suggested it provides a feasible inverse design selection for wave control structures that can be utilized for acoustic corner/carpet cloaks. Similar databases can be greatly beneficial for future acoustic cloak design and specific requirements.

Acoustic cloaks via scattering cancellation^103^ have also become a topic of interest due to their robust designs, operating spectral range, and fast fabrication. In such schemes, isotropic layers of a specific thickness, mass density, and bulk modulus can be carefully tailored to cancel the first few scattering orders, which significantly reduces the scattering cross-section of the system, to make the object nearly undetectable at a particular frequency. Ahmed et al. have designed an ML-driven acoustic invisibility cloak with a multilayered core–shell configuration.^104^ A probabilistic deep DL model was utilized based on an autoencoder-like NN. The network successfully selected structural and material combinations of the cloaking core–shell and suppressed sound scattering (Figure 3B). Similarly, Tran et al. have presented a DL framework for predicting the optimal scattering metamaterial for invisibility in different wavenumbers.^105^ The planar configuration of scatterers within the metamaterial is usually used to block this scatter and obtain optimal cloaking effects. Total scattering cross-section (TSCS) must be taken into consideration to design a planar configuration. The group developed an artificial NN with probabilistic generative modeling and DL with both supervised and unsupervised models. The framework created cloaking metamaterial with minimized TSCS within minutes, which could be considered “express delivery” in contrast to traditional methods. Several studies have also demonstrated the feasibility of DNN for acoustic cloak design due to the fast iteration ability (Figure 3E)^106,107^ under different conditions such as water and air^108^ as well as for the development of a biphysical cloak with triple-wave cloaking capabilities.^109^

One challenge in this domain is the manufacturing of AI-based acoustic cloak designs. Simple designs can be fabricated by conventional fabrication techniques such as casting, injection molding, laser cutting, and casting.^110^ On the other hand, complicated 3D structures such as channels^111^ or chambers,^112^ mainly relying on semi/manual manufacturing. Yet, manual manufacturing comes with a lower consistency and high standard deviation of product quality. In some designs, new manufacturing technology such as 3D printing can be utilized, however, the same challenges of low-production efficiency and limited material selection remain. Therefore, the inclusion of manufacturability parameters in AI models should be prioritized, whenever possible, to better explore new generation metamaterials and designs that can be compatible with current process lines.^113^

## 3 Emerging Applications of AI-Based Optic Metamaterial Design

The discipline of optic metamaterials (OM), which involves the engineering of materials to control or manipulate optic properties, is undergoing a transition with the integration of AI and ML. The design of OM comes with two main challenges: design bottlenecks and manufacturability bottlenecks. First, the design process involves computationally intensive tasks,^114^ intricate geometries,^115^ and diverse optical response analyses.^116^ These complexities can pose deviations for achieving the desired optical response. Second, OM can be highly sensitive to small variations in meta-atom arrays and their units. The sensitivity deteriorates calibration, tuning, and manufacturability.^117^ At this point, AI-based design methodologies are transforming the field via computational models and ML algorithms since these strategies can uncover patterns, optimize designs, predict the optic performance of novel materials, and analyze different variations. In recent years there have been groups exploring AI and AI-assisted design for meta lenses, beam splitting, and meta-grating among other applications.^118−122^

### 3.1 Optic Meta-lens

**Figure 4:**
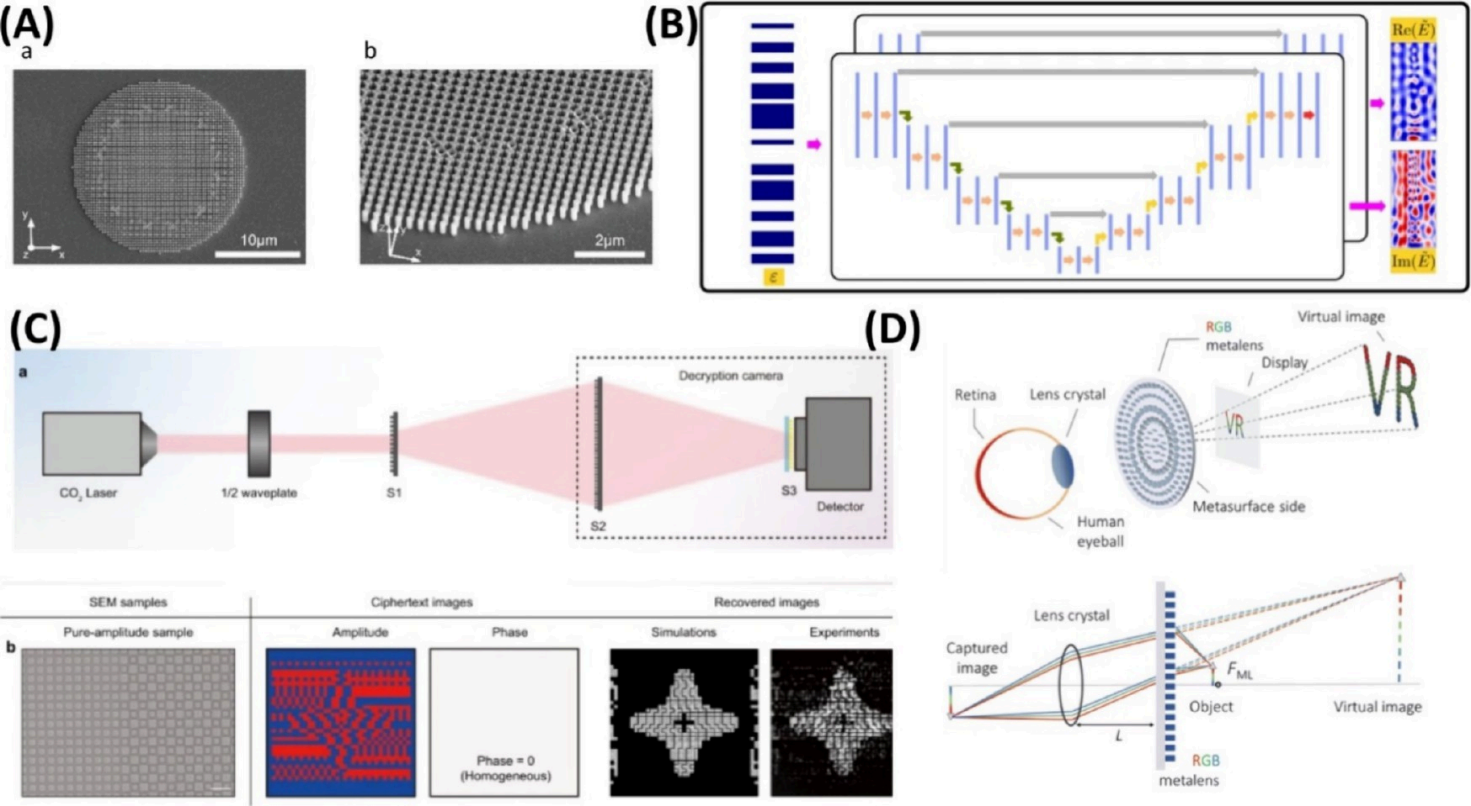
Optic metamaterial and AI-based design. (A) Visible achromatic metalens design. Adapted with permission from ref (139). Copyright 2022 Wiley. (B) Extensive area optimization of the optic lens via data-free ML and its decision network. Adapted from ref (129) in accordance with its Creative Commons CC BY license. (C) Meta-optic empowered vector visual security. Adapted from ref (125) in accordance with its Creative Commons CC BY license. (D) Meta optic for virtual reality and demonstration via alphabet. Adapted from ref (44) in accordance with its CC BY-NC 4.0 license.

Meta-lenses use the interaction of light and the meta-atom with a specific three-dimensional geometry. The interaction presents an optical response by an array of meta-atoms on the metamaterial. These arrays can alter wavefronts such as phase and amplitude with desired optical responses. Capable of manipulating the phase distribution, optic lenses are used in the most practical applications.^123,124^ The applications (Figure 4D) include virtual reality technology^44,45^ and security encryption (Figure 4C).^125^ AI-based design methodologies transform the field via computational models and ML algorithms. Some of the meta-lens design approaches include genetic algorithms,^126−128^ global optimization,^129^ local search,^130^ and saddle point construction.^131^ Although useful, the mentioned methods still can have difficulty reproducing and ensuring optimal design, accuracy, and adaptability.^132^ Thus, ML and artificial intelligence approaches have gained widespread attention.

Multifunctional optic lenses resort to accommodating multifunctionalities at the cost of increased structural complexity. This can enable the development of a “Swiss knife” but comes with intrinsic design restrictions due to the cost of investigation of the meta-atoms. There have been several reports to address this bottleneck without referring to traditional phase retrieval and meta-atom structural design. Ma et al. have proposed an embedded ML model for the automatic implementation of multifunctional surfaces^133^ and demonstrated this via hologram and lens focusing in the near-infrared region. In contrast to traditional methods, the proposed framework facilitated a prescribed design space. The group utilized gradient-based and nongradient optimization loops to implement multifunctional metasurfaces automatically. A single-layer focusing lens in the near-infrared region with eight controllable responses subjected to different combinations of working frequencies and linear polarization states was developed. Similar capability and automatization via the data-driven scheme for optical lens design were presented with the transition to NN.^127,134−136^

Surrogate models are commonly used in the optic lens design to evaluate components. They can utilize partial differential equations rapidly but come with a training cost of multiple variables. In optic metalens design, training costs can increase abruptly for larger areas.^137^ As a result of this bottleneck, acceleration and optimization of its design have gained attention.^138^ Pestourie et al. have presented an active-learning algorithm to accelerate design time by at least 1 order of magnitude. Furthermore, the simulation time was reduced by at least 2 orders of magnitude compared to that of uniform random samples. The approach selected the training points based on the error measure and utilized an NN surrogate model for partial differential equations (PDE). For instance, optic metalenses capable of converging light at three wavelengths into three different focal spots were developed.^138^ Similarly, a backpropagation NN-based tool for designing high-performance optic metalens with accelerated simulation was generated. A reservoir of phase modulation data was formed in seconds via the NN, and the model was able to generate thousands of meta-atoms. The NN was developed by designing meta-lens with achromatic focusing, imaging within a visible wavelength (420–640 nm), and with no polarization dependence.^139^ Although the time acceleration is desired for the design timeline (Figure 4A), the accuracy and tolerance of the intelligent component must be considered and preserved.

Other examples of recent DNN-based applications in optic metalenses have encompassed a diverse range and include the development of chiral metasurface multifocal lens in the Terahertz band^140^ and miniaturized wide-angle fisheye lens^141^ among other applications.^142,143^ Wang et al. have designed bifocal metalens that can independently focus and its bidirectional circular polarized light. In the traditional sense, simulations for meta-lens design have a high computational cost thus, the group suggested a DL-forward genetic algorithm to design the metalens parameters efficiently. The design presented the flexibility to change the intensity ratio of lens focus via manipulating incident light ellipticity. Furthermore, it did not require redesigning the light-intensity profile.^144^ A similar approach with a multifunctional metasurface device was reported by other groups.^145,146^ Although there have been many studies, research transferability has remained limited and bottlenecks for computational costs and automatization for different designs have not yet been fully addressed. In addition to these, data availability is typically a prerequisite to train AI models in metamaterial design, with few exceptions. Zhelyeznyakov et al. have presented a more extensive area optimization (Figure 4B) of meta-lens via data-free ML.^129^ A similar RNN has been designed by Valantina et al. for coherent Lightwave scattering on the millimeter scale in deep-tissue microscopy. However, the group did not demonstrate any inverse design.^147^

### 3.2 Meta-grating

In photonics, complex on-chip components are essential to manipulate light waves. Some optical metamaterials utilize subwavelength meta-atoms allowing scientists to sculpt different light propagation patterns. This plays a crucial role in beam engineering in integrated photonic applications such as grating and has been used for applications of infrared, Raman, and spectroscopic analysis.^148^ During the meta-grating design, traditional optimization methods can fail to capture global optimum with a feasible process. Data-driven and AI-based solutions are emerging tools to address these issues.^149^ Singh et al. have presented a CNN and DNN for the inverse design of optical dielectric metamaterials for integrated photonic applications. The feedforward DNN and CNN architecture predicted the repetition period, height, and size of the scatters. It outperformed conventional (non/gradient descent-based, genetic) optimization approaches regarding design time, and predicted the diffraction profile with a correlation coefficient of 0.996.^150^ The obtained design was compatible with the grating of different beam profiles (uniform, focused, or Gaussian). For the design of the meta-grating structure, DNN performed better than the CNN-based function estimator for the given training set. It was demonstrated that in contrast to time-intensive iterative design approaches, NN allows more rapid estimation of design parameters via a free space diffraction profile. Another group reported a Tandem-structured DNN to design a grating meta-atom, five-layered metal–insulator. The trained DNN was able to learn physical knowledge from data and facilitated grating structures that had an average mean squared error (MSE) of 0.023. The group additionally fed spectral information on resonant wavelengths and reflection spectrum to DNN and the DNN was able to design for gradually changing target wavelengths.^122^

The generality of DNN/CNN was implemented by other researchers and its potential benefits for fabrication were noted since it can be adapted to design complex lab-on-chip meta-graters with high-dimension design spaces.^151−154^ A recent study by Juodenas et al. presented an on-chip illumination device with curved gallium arsenide (GaAs) meta gratings integrated on vertical cavity surface emitting lasers (VCSEL). The illumination system was capable of total internal reflection and dark field microscopy via rapid switching.^155^ Flat meta-optics have been replacing classical optics elements and can help to design compact biphotonic devices within lab-on-chip. Shaping the light into wide angular range wavefronts with high efficiency is a challenge such as in high-contrast microscopy applications. The group provided an alternative illumination solution for high-contrast imaging. The systems can be transferable and integrable from portable microscopy, NIR-II range bioimaging, and lab-on-a-chip devices. The mentioned examples showcase the application of AI to bioimaging and lab-on-chip. There are still bottlenecks of miniaturization and feasibility concerns.^156^ These concerns can potentially be addressed by simulations which could have a considerable amount of computational cost. The simulation requirement in computation resources has been reduced with the assistance of ML algorithms such as NNs in previous cases.^157,158^ Overall, such strategies could help address these issues.

## 4 Emerging Applications of AI-Based Metamaterial Design in Healthcare

The healthcare field is undergoing a transition with the integration of AI and ML.^159^ With the growing population, disease monitoring,^160,161^ tissue engineering,^162,163^ and diagnostics^164,165^ have become increasingly important to ensure public health and advance person-centered healthcare. The growing demand for accurate diagnostic technologies and health monitors can be met via AI-based design.^166^ Novel or tailored metamaterials have great potential to meet the specific demands of healthcare settings and can be used for vital sign monitoring, the detection of biomarkers, and the development of movement sensors.^167−170^ In healthcare, data availability and quality are crucial to ensure adequate treatment and prevent complications. Early detection of markers as well as continuous monitoring can help reduce complications and decrease mortality. Thus, the development of accurate and sensitive sensors is crucial in the field of medical diagnostics. To this end, AI models can be utilized in various ways. For instance, medical data can be used to train AI models to enable models to understand complex relationships between target markers and sensors. In contrast to other fields, data accessibility might be more restricted since medical data and patient privacy are subject to international and local laws.^171^ Such regulations are essential to safeguard patient rights. While high-quality data is often required to train AI models, some models have been developed using limited and/or unstructured medical data.^172,173^ Alternatively, AI-based design can be employed to develop diagnostic sensors or healthcare monitors with improved functionalities.^1,174^ Moreover, several groups have reported AI-based metamaterial design in healthcare.

### 4.1 Diagnostics Sensors

The inclusion of terahertz metamaterial absorbers (TMA) is a promising real-time and sensitive platform for detecting biomarkers and microorganisms.^175−178^ They are designed to utilize terahertz radiation and dielectric sensing by obtaining information via the dielectric constant of the target sample.^179−182^ Other biosensing systems include mechanical sensing^183,184^ and resonator or electrical biosensing.^185^ These types of biosensors rely on the target biomarker attachment to a mechanical resonator or within a set radius of an electrical circuit where it can be degraded.^186^ The TMA concept has gained attention as it is noninvasive and relatively nondestructive. The noninvasive nature allows repeated measurement of samples with minimum damage. Additionally, THz waves are lower energy waves than X-rays and γ rays therefore making it relatively safer for the patient or operator.^186^ However, the system has some drawbacks such as higher power consumption, sensitivity to environmental changes, complex design optimizations, and fabrication challenges.^187,188^ There are also certain bottlenecks for material optimization, scalability, and the effective translation of laboratory innovations into practical biomedical applications. AI models can be utilized to comprehend and predict interactions between absorption values for combinations of variant wavelength values, substrate thickness, graphene potential, and resonator thickness values. Thus, AI-based models can enhance the design and simulation timeline. It can make TMA more feasible for use in the healthcare landscape.

**Figure 5:**
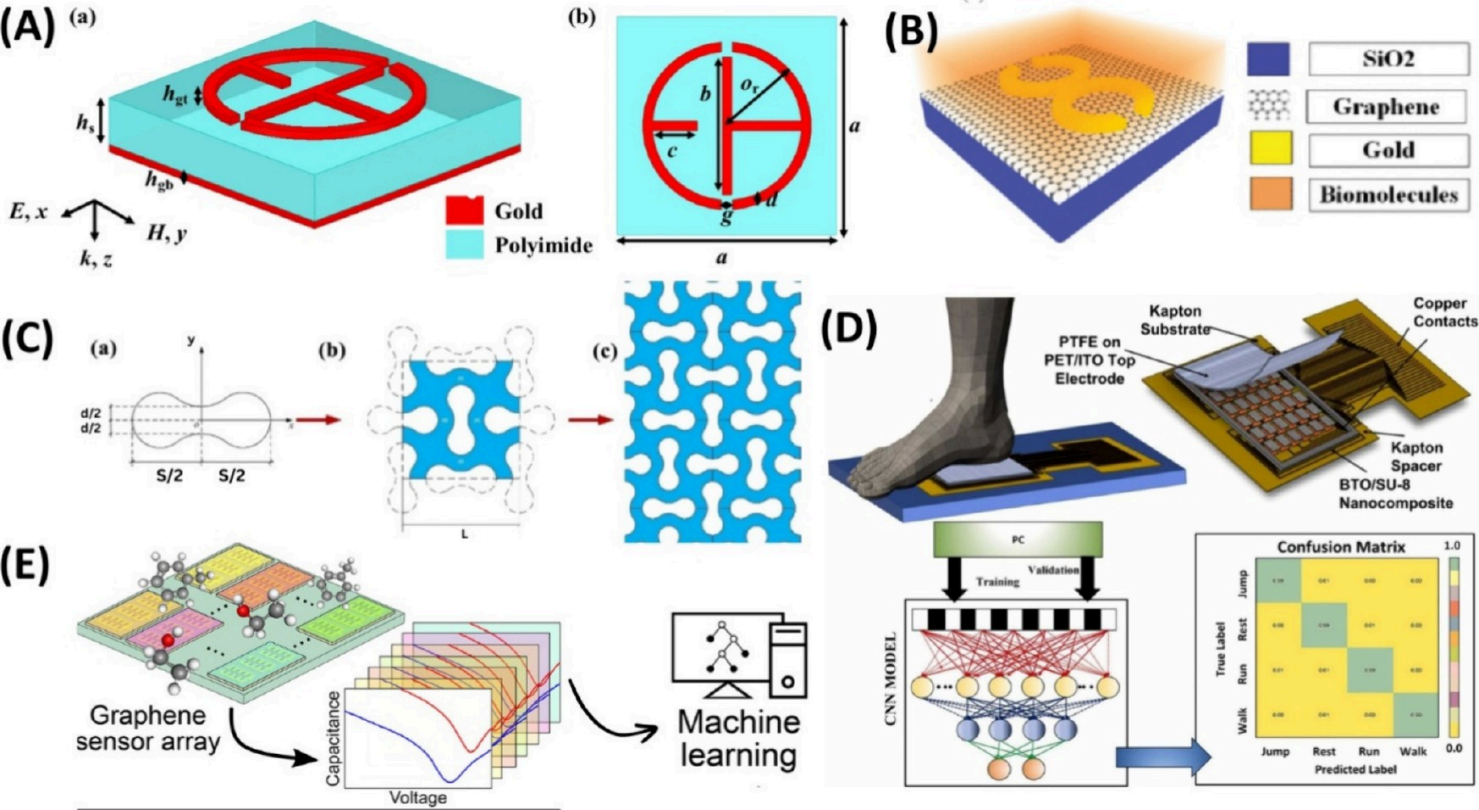
AI-based metamaterial design in healthcare. (A) ML assisted hepta band THz metamaterial absorber for biomedical applications. Perspective view and Top view of the proposed metamaterial absorber. Adapted from ref (121) in accordance with its Creative Commons CC BY license. (B) Reversible intelligent design for perforated auxetic metamaterials with a peanut-shaped pore, b unit cell, and c plane array of unit cells. Adapted with permission from ref (243). Copyright 2023 Springer. (C) 3D view of the graphene-based single split ring resonator (GSSRR) design, Adapted with permission from ref (189). Copyright 2023 IEEE. (D) ML-assisted hybrid transduction nanocomposite-based flexible pressure sensor for gait analysis. Adapted with permission from ref (239). Copyright 2023 Elsevier. (E) ML-based rapid detection of volatile organic compounds in a graphene electronic nose. Adapted with permission from ref (231). Copyright 2022 American Chemical Society.

One group has designed a graphene-based metasurface refractive index biosensor for hemoglobin detection. A polynomial regression (PR) model was employed to predict the absorption values for combinations of variant wavelength values, substrate thickness, graphene potential, and resonator thickness values.^189^ The adjusted R2 score was close to 1.0 at a higher (>5) polynomial degree, which indicated a relatively high prediction efficiency for a regression model (Figure 5C). An accurate hemoglobin sensor can be crucial to assess disease progress, healthy tissue, and blood vessels. Moreover, hemoglobin and hemoglobin oxygenation are vital biomarkers in various diseases.^190−192^ In many cases, changes in the oxygenation of organs or tissues can indicate the presence of a trauma injury, or illness, such as diabetes, obstructive pulmonary disease, or cancer.^193,194^ Low oxygenation or low perfusion can also show tissue viability and the extent of potentially irreversible organ or tissue damage. Besides oxygenation sensors, similar graphene-based sensors have been reported in immunosensing,^195^ cancer cell detection,^196^ and viral genome detection.^197^ Another group, Jain et al. have designed a hepta-band terahertz metamaterial absorber (MMA) with the highest sensitivity of 4.72 THz/RIU for glucose detection via the extreme randomized tree (ERT) model. The impedance matching theory and electric field distribution were utilized via the Extreme Randomized Tree (ERT) model to predict absorptivity for intermediate frequencies with unit cell dimensions, substrate thickness, angle variation, and refractive index values to reduce simulation time. A modified dual T-shaped resonator on polyimide was deposited on MMA with ultrathin (0.061 λ) and multiple absorption peaks (Figure 5A). The ERT model in predicting absorption values was evaluated using the Adjusted R2 score, close to 1.0, which can indicate good prediction efficiency in cases. Furthermore, the simulation time was cut down to 60%. The outcome showed computational sources can be saved by simulating absorber design using the ERT model. The group also suggested that the model and proposed sensor had transferable applications in the biomedical field for malaria detection.^198^ This system can potentially be helpful for fast-track patient care in crowded or rural hospital settings and in areas where access to medical care is limited.

AI-based metamaterial design demonstrates amplified practical engineering for diagnostics. The approach presents capabilities such as increased sensitivity, accelerated detection time, or convenience of continuous monitoring. One example of practicality can be the label-free detection of biomarkers. Cost-effectiveness, ease of use for users, low sample volume, and multiplex detection are some advantages of label-free biosensors over traditional biosensors.^199−201^ The long-term durability and biocompatibility of AI-designed metamaterials add to their potential to transform diagnostics. The forecasts can be achieved using AI, together with the advent of Medical Internet of Things (M-IoT) devices and can pave the way for significant advances. Prioritizing the development of metamaterials with real-time monitoring and noninvasive detection is consistent with the growing need for M-IoT devices. AI models, particularly designed for optic metamaterials capable of detecting biomarkers in saliva or sweat, demonstrate possible advancements in patient-centered and no-pain diagnostics.^202−204^ Investigating the combination of cellphones and plasmonic devices, making use of their light sources, cameras, image processing, and communication capabilities, can lower expenses and enable widespread dissemination of these cutting-edge diagnostic tools. All things considered, the AI-driven diagnostics design, based on metamaterials, has the potential to diagnose and monitor patient’s daily life.^205−207^ As such, AI-based metamaterials are rapidly redefining the commercialization potential for diagnostic tools. Determining a metamaterial’s performance, longevity, and appropriateness for a given application requires precise metamaterial property prediction. The accuracy and half-life of these novel metamaterials become crucial to prevent liability concerns. Although briefly mentioned, the issue of product liability has not yet been thoroughly discussed in the context of inverse/forward design.^208,209^

### 4.2 Point-of-Care Devices

AI-based design has been utilized to improve the capabilities of metamaterial-based wearable sensors in point-of-care settings (POC).^1,210−213^ In the past decade, M-IoT devices have been widely accepted and used to record medical data on vital signs and biomarkers. The acceptance of M-IoT devices has drawn attention to the development of wearable sensors and their physiological data records. In the past decade, examples of electronic tattoos,^214^ biofluidic wearable sensors,^215^ and textile sensors^216^ have been introduced as emerging applications of metamaterials. Current applications are limited by the inherent properties of materials, their quality, biocompatibility, and fabrication bottlenecks. The intrinsic structure and functionality of metamaterial can be improved using AI-based or integrated solutions.^217^

Active research of AI-based metamaterial design is chiral plasmonic metamaterials in wearables.^218^ In the last decades, AI and high-performance computing (HPC) have enabled more feasible computational costs.^219^ There have been several reports of DL implementation for biomolecular enantiomer sensing.^220−223^ Yang et al. have reported a wearable plasmonic sensor based on flexible plasmonic metamaterials with surface-enhanced Raman scattering (SERS).^224^ The sensor had a superlattice metafilm structure that is prone to deformations and thus can potentially alter SERS activity. To maintain SERS activity, the group proposed an “interconnected island” design with a small guard ring. The ring supported the metafilm and prevented the deformation of the SERS activity center. FEM-based stress analysis was conducted before plasmonic metamaterial design to understand the deformation risks and structure optimization. The proof-of-concept was demonstrated with a nicotine/sweat case study. The outcome showed its potential for use as a continuous monitoring tool. The use of AI-based design of chiral plasmonic metamaterial has also been explored in prior studies aiming to develop personalized POC applications.^225−227^ Such applications include DNA sensing,^228^ glucose quantification,^229^ bacteria detection,^230^ and the development of an electronic nose (Figure 5E).^231^

The advantage of AI is not only restricted to design but also to fast analysis of medical data. This can be beneficial, especially in a hospital setting to fast-track patient care or improve time-dependent analysis.^41^ Xie et al. have reported an FC-NN model for analyzing the CD response of chiral plasmonic metamaterials.^232^ A permutation importance method (PIM) analysis was utilized to detect which parameters have the largest effect on CD response. The CD response has a direct implication for the metamaterial structure thus, on functionality and manufacturing. The group suggested extracting certain intervals of the CD spectral line: the peak magnitude of CD and the corresponding wavelength information. The suggestion was fed to permutation importance response and determined to be useful. The network structure of the model was simplified while improving the prediction accuracy of the peak magnitude of CD. The model was able to refer to smaller data sets and still maintain prediction quality. The prioritization of CD data and PIM might be beneficial as it can lower computational costs and data storage and enable system optimization. Despite these reports, the potential of plasmonic metamaterials in POC healthcare devices is yet to be fully established.

Another emerging field of POC and metamaterials is wearable tactile sensors. During the sensor design flexibility is a desired characteristic to maximize patient comfort, optimize wearable device design, and ensure stretchability.^232^ This design preference is usually supplemented via stretchable, flexible, or auxetic metamaterials to be compatible with the user and their movement. In a POC setting, wearable tactile sensors have a wide range of functions including pressure, strain, shear, and vibration parameters. In this regard, tactile wearable sensors can be used to improve the quality of life of certain patient groups. By integrating AI and computational strategies, higher functionality can be achieved for wearable metamaterial sensors and their design process.^233^ Alternatively, a combination of metamaterials can also enable variant design requirements with elastic properties. In recent years, AI-based design has emerged in auxetic metamaterials in relevance to tactile sensors.^234−237^ For example, Nadeem et al. have reported an ML-assisted flexible pressure sensor for human gait analysis (Figure 5D).^238^ The gait analysis is a common physiological well-being test and correlates with critical health metrics. Accurate and cost-effective gait monitoring can be life-saving in several medical disorders.^238^ The designed sensor had flexible hybrid transduction Barium Titanate (BTO)/SU-8 nanocomposite with a pressure sensor matrix. Contrary to other sensors, the group utilized a 36-pressure cell with hybrid transduction. The sensor could deliver rich feature extraction to ML algorithms compared to single transducer-based systems in gait and grip strength monitoring. The integrated CNN-2D model demonstrated an accuracy of 98.5% for gait characterizations.^239^ Similarly, Wu et al. proposed a FEM-based metamaterial design for improved sensitivity and durability. The device was composed of a wire strain sensor with six auxetic units and showed high sensitivity (GF = 21.8 at ∼80–130% strain) and high stretchability (130%).^240^ This system could potentially be helpful to fast-track patient care in crowded hospital settings and access to medical care in nonprivileged demographics.

In the field of auxetic metamaterials, the perforated metamaterials with peanut-shaped pores display the additional advantage of flexibly tunable mechanical characteristics. One challenge is to model them through conventional physics methods while targeting various auxetic requirements. Since it is usually time-consuming to achieve this,^241,242^ Liu et al. have demonstrated a hybrid model via the coupling of a back-propagation neural network (BPNN) and genetic algorithm (GA).^243^ Microstructure–property pairs were utilized to train BPNN and predict the relationship of microstructural parameters meeting the target Poisson’s ratio (Figure 5B). The group verified the accuracy of the model via finite element simulations. They were able to accelerate design under constrained/unconstrained conditions. This demonstrated, to some extent, that AI-based design can accelerate the design of flexible metamaterials with high low-stress concentration levels for lower fatigue within the structure. Other examples of auxetic metamaterials in POC include the development of prosthetic electronic skin,^244^ sign language translation via gloves,^245^ bioinspired tactile nociceptor for mimicking sensitization phenomena,^246^ electronic textiles,^247^ and medical linear accelerator.^248^ Such applications of metamaterials hold great potential to improve users’ daily lives and to provide better health monitoring.

## 5 Emerging Applications of AI-Based Metamaterial Design in Power Harvesting and Transfer

Traditionally, batteries have served as the primary energy source for wearable and portable devices, yet their inherent limitations in terms of size, weight, and disposal create significant challenges; however, as low-power electronic design advances, there is a burgeoning opportunity to harness energy from the environment through techniques known as Energy Harvesting (EH), enabling direct power supply to electronics or secondary battery recharging.^249^ Waves, such as vibration, sound, and light, interact uniquely with matter, but a significant portion of their energy is dissipated through processes like material damping and friction, prompting research into ecologically benign energy sources that efficiently harvest and convert this waste energy into electricity, with the choice of transducers dependent on the specific wave type and conversion method.^250^ Conversion efficiency, a crucial factor in EH devices, is heavily influenced by the choice of conversion medium, and while natural materials were historically favored, their limitations in terms of efficiency stem from inherent material properties and structures. Recent years have witnessed the immense potential of artificial materials and structures, especially metamaterials, with their unprecedented physical properties such as negative stiffness, mass, Poisson’s ratio, and refractive index, which are absent in natural materials, leading to innovative opportunities in EH through nontraditional physical behaviors.

**Figure 6:**
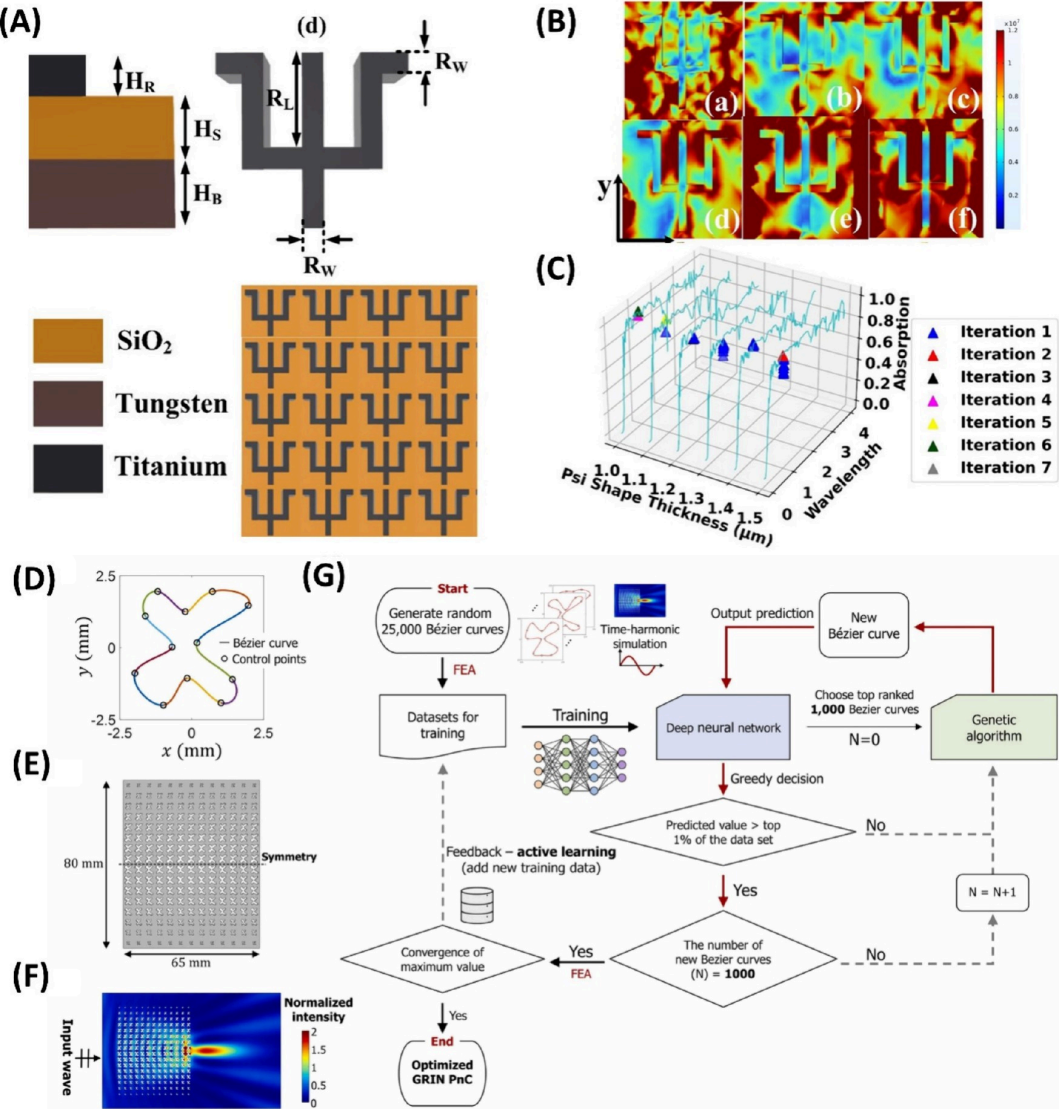
(A) Frontal perspective of the suggested absorber structure, magnified view of the psi-shaped resonator, and periodic array illustrating a unit cell of the proposed psi-shaped solar energy absorber. The dimensional parameters include tungsten base layer thickness (*H*_B_), silicon dioxide (SiO_2_) substrate thickness (*H*_S_), the titanium psi-shaped resonator thickness (*H*_R_), psi shape’s width (*R*_L_) and length (*R*_W_). (B) Electric field intensity calculated within the *x*–*y* plane (a–f) for various wavelengths. (C) A scatterplot shows the state absorption during random restart hill climbing, which was employed to identify the optimal psi shape thickness. (D) Cubic Bézier curves that consist of random input (control) points placed in a piecewise manner. (E) Arrangement of the GRIN lens, with the dashed line indicating the symmetry axis. (F) The normalized two-dimensional intensity plot relative to the focused intensity of a circular GRIN lens. (G) Flowchart outlining the design optimization process based on active learning. Subfigures (A–C) are reproduced with permission from ref (252). Copyright 2022 Wiley. Subfigures (D–G) are reproduced with permission from ref (303). Copyright 2022 Elsevier.

Researchers have discovered that metamaterials could be utilized as solar thermal energy absorbers due to their broadband absorption properties.^251^ In a study by Patel et al., a psi-shaped solar energy absorber was examined, which was computationally modeled using the FEM technique and optimized using an AI-based method.^252^ The group emphasized the significance of a broad-spectrum solar energy absorber to enhance the absorption range of a solar thermal system, highlighting the need for efficient solar radiation absorption across a wide range of wavelengths.^252−254^ They designed their assembly by initially using a base layer of tungsten (W) metal, followed by placing a silicon dioxide (SiO2) substrate on top and positioning a psi-shaped resonator made of titanium (Ti) material over the substrate. The design parameters included specific dimensions for the structure and its components (Figure 6A), which were numerically simulated using COMSOL Multiphysics, considering a range of planar light wavelengths to calculate the electric field intensity (Figure 6B) and the energy absorption of the metamaterial. Next, the AI-based random restart hill climbing (RRHC) technique was employed to design experiments aimed at identifying the optimal tuning parameters for metamaterial solar absorber design, utilizing a predefined set of experimental design parameters (Figure 6C). This study demonstrated that the developed structure achieved an absorption rate exceeding 90% and significantly reduced computing power and time requirements for the simulation process by 97.57% because of the optimization process using the RRHC technique.

In another study by Lee et al., gradient-index (GRIN) phononic crystals (PnCs) were optimized using an ML-based approach aimed at enhancing energy harvesting.^7^ PnCs are artificially engineered materials, featuring periodically distributed inhomogeneities, and were actively researched for their capacity to manipulate the propagation of input acoustic/elastic waves to localize or focus energy, thereby amplifying output harvesting performance,^255−257^ and particularly GRIN PnCs served as a lens equivalent for acoustic/elastic waves.^250,258−262^ Using commercial COMSOL code, they designed unit cells with 12 random control points on a cubic Bezier curve (Figure 6D) and constructed a GRIN lens measuring 13 units in width and 9 units in height using the generated unit cell to establish a gradient in a hole size (Figure 6E). They then calculated the focus intensity of a 1000 mm × 2000 mm aluminum GRIN PnC plate (Figure 6F). Then, 25 000 random Bezier curves were created for the training data set via a DNN with 13 hidden layers, each of which containing 24 neurons. The top 1,000 best-performing configurations were selected to be used in Genetic Optimization (GO) to produce new Bezier curves, and finally find the optimized GRIN PnC. Figure 6G illustrates the flowchart of the active learning-based optimization process. The GRIN PnC structures were manufactured in a 2 mm thick aluminum plate through precision drilling machining according to the optimized design, for experimental validation purposes. The AI-based GRIN PnC design demonstrated 1.35 times greater wave energy focusing than the previously used circular GRIN PnC model. The authors also mentioned that the experimental and simulation results do not perfectly align, and this mismatch can be attributed to several factors, including differences in incident wave generation and potential material imperfections such as residual stress near the GRIN hole.

**Figure 7:**
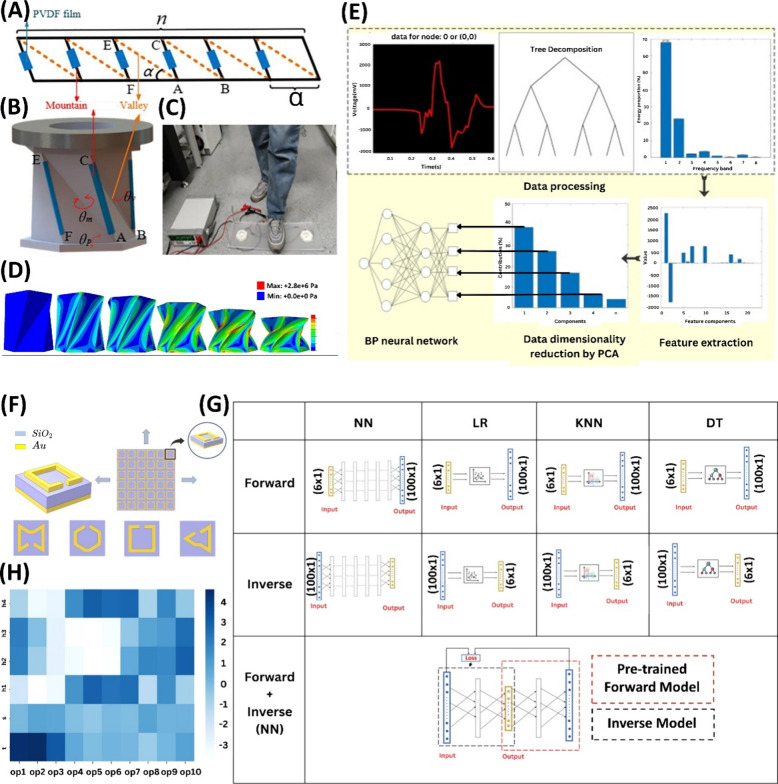
(A) The crease pattern of the Kresling origami; a dotted line depicts the valley crease, and a solid line represents the mountain crease, with the PVDF piezoelectric film marked by a blue rectangle along the mountain crease. (B) The tangible representation of the piezoelectric Kresling origami generator, with specified folding dihedral angles: θm for the mountain fold, θv for the valley fold, and θp for the bottom fold. (C) Demonstration of the generation of open-circuit voltage data experimentally. (D) Finite element outcomes depicting the distribution diagram of Mises stress during the quasi-static compression of the Kresling origami structure. (E) Flowchart outlining the training process for the backpropagation NN, involving voltage data decomposition using a three-layer wavelet packet, signal group formation based on energy ratios, derivation of 22 characteristic data sets, extraction of the top four principal components using principal component analysis, and their utilization as input in a double-layer NN for training and testing. (F) A 3D perspective of a metamaterial unit cell composed of gold layers on top and bottom, separated by a silicon dioxide middle layer, and a 2D view demonstrating different unit cell designs achieved through structural parameter modifications. (G) The structure of all the models, including the forward model for absorption spectrum prediction from structures, the inverse model for predicting structures from absorption spectrum, and an inverse NN model with a tailored loss function. (H) A correlation heatmap displaying the relationship between input structural parameters on the vertical axis and 10 predicted values on the horizontal axis, where each predicted value represents the average of 10 output points (out of 100) as utilized in the forward model. Subfigures (A–E) are reproduced with permission from Elsevier.^263^ (F–H) Subfigures are reproduced with permission from the American Chemical Society.^51^

Another research by Huang et al. introduced a self-powered biometric device, featuring a Kresling origami metamaterial and piezoelectric energy harvesting technology, to achieve the conversion of energy from trampling actions and the identification of trampling objects.^263^ The piezoelectric Kresling origami metamaterial is an innovative energy generator that merges Kresling origami with PVDF piezoelectric films. The mechanical model employed a single degree of freedom forced vibration model, and the dynamic governing equation was established by determining the nonlinear support reaction force during the deformation of the Kresling structure (Figure 7A–C), with particular attention to the elastic potential energy stored mainly at the crease of the structure. Finite element simulations, sample production, and quasi-static compressive experiments were conducted on the Kresling origami structure (Figure 7D). The Kresling origami structure employed specific geometric parameters with several sides of a bottom regular polygon of 6, an angle of 52°, length of the bottom edge of 35 mm, crease thickness of 0.8 mm, crease width of 0.002 m, crease length of 0.045 m, and Young’s modulus of 45 MPa, and an external load was applied to compress the upper surface downward by 25 mm. The Kresling origami sample, composed of Thermoplastic polyurethane (TPU), was manufactured in a three-step process, involving mold design and fabrication, heating and melting of TPU material within the mold, and demolding once the sample was shaped. Three students, with varying weights, were chosen to assess the device’s performance and conducted a combined 110 passes through the pedal using both their right and left feet, collecting gait data for training and testing the device’s performance, revealing variations in generated voltages based on individual interactions. The authors reported that the challenge for training data was a small number of data sets in this study, and in response, they used a ratio of 10:1 for division to ensure ample training data. ANN model with two hidden layers is trained using 600 data sets, while the remaining 60 data sets serve as a test set (Figure 7E). As anticipated, the results indicate that individuals with greater weight generate higher output voltage due to increased Kresling deformation, and they also reveal a 100% recognition accuracy for all three individuals based on the NN model.

In another research by Soni et al., the authors demonstrated an AI-driven framework for optimizing terahertz-range metamaterial absorbers, which also involved the development of ML models to predict absorption spectra based on structure and vice versa.^51^ Metamaterial absorbers (MMAs) designed for operation in the terahertz range (spanning from 0.1 to 10 THz) garnered substantial research attention due to their diverse applications including energy harvesting.^51,264^ The research employed COMSOL Multiphysics’s wave optics module to simulate the proposed metamaterial perfect absorber (Figure 7F), which featured ten structural parameters, six of which were variables, encompassing four height parameters (h1, h2, h3, and h4), split-ring thickness (t), and split dimension (s). The simulations, operating in the frequency domain with Maxwell’s equations, established the relationship between electric and magnetic fields and their sources to gain insights into electromagnetic wave behavior, generating absorbance plots for about 6000 structural parameter configurations of the metamaterial structure. Among the ML models, K-nearest neighbors (KNN), linear regression, decision trees, and NNs were employed for both the forward and reverse models (Figure 7G). In the forward model, structural parameters served as input, the absorption spectrum as output, with K-nearest neighbors and decision trees implemented using Scikit-learn and NNs using TensorFlow, comprising an input layer with six nodes, an output layer with a hundred nodes, and five hidden layers with 64, 128, 512, 1024, and 512 nodes, respectively, while model performance was evaluated through mean squared error (MSE). Two types of inverse NN models were employed, one using a default loss function and the other incorporating a custom loss function that utilized the forward model, both featuring a common architecture with an input layer of a hundred nodes, an output layer with six nodes, and five hidden layers of varying node counts and in the case of the NN with the custom loss function, this function comprised two components: one involved passing the inverse model’s output through a pretrained forward model and calculating the mean squared error, while the other component consisted of the standard mean squared error, constituting the overall custom loss function used for model training. To ensure impartiality in the model, a correlation heatmap was constructed, depicting the relationships between the input factors and the output results (average absorption values for every ten data points) using correlation coefficients to gauge their linear associations (Figure 7H). The performance of both the forward and reverse models was assessed, revealing that the best results were achieved by the NN model employing the default loss function, with errors of 0.0028 for the forward model and 0.023 for the reverse model, and visual representations were included to validate the predictions.

## 6 Current Challenges and Future Trends

AI-based design has been utilized in metamaterials for over a decade. ML techniques such as Gaussian and gradient boosting are commonly used for topology and microstructure data predictions. In comparison, NNs are used to improve the accuracy, time, and generation of novel metamaterials via generative models such as Generative adversarial networks and variational autoencoders (VAEs). They are powerful tools to predict, simulate, and design the desired functionality of the metamaterial. Acceleration of the design process opens new possibilities for applications such as solving process bottlenecks, overcoming human design limitations, increasing the half-life of metamaterials, and enhancing specific mechanical properties. On the other hand, there are still gaps in the design capabilities of AI such as achieving the complexity of mechanical properties for the desired function, addressing safety regulations, ensuring prediction accuracy, maintaining data accuracy, handling big data, optimizing AI models, implementing data security, and allowing transferability/generalization (Figure 1B and Table 3).

**Table 3:** Field of Healthcare, Acoustics, Power Transfer and Harvesting, and Optic Metamaterials: Their Current Bottlenecks and Future Trends

	Current bottlenecks	Future trends
Healthcare	Handling health data	DL-based prediction^265^
	Data privacy	Model privacy^266^
	Ethical regulations	Cloud systems and security^267^
	Biased output	Data encryption and differential privacy^268^
	Liability	Validation, international, and internal audit^269^
Acoustics	Tunability	Multiple-input multiple-output (MIMO) systems
	Half-life	Generative models for novel metamaterials^270^
	Fatigue	Healing metamaterials^271^
Power harvesting and transfer	Transferability	Multitask DL-based design
	Generalization	Additive manufacturing^272^
	Power security	Generative design for sustainability
	Manufacturability	Simulations
Optics	High error rate	Deep learning^273^
	Computing	Optical neural networks^274^
	Handling big data	Cloud systems^267^

### 6.1 Handling Big Data

AI models need efficient handling and enormous data to generate accurate predictions. The outcome quality is usually proportional to the data set quality such as format, consistency, duplication, integrity, accuracy, and completeness. Due to this prerequisite, AI-based design might have bottlenecks in processing and feasibility of big data handling, data preparation, and back-upping.^275,276^ Large data or unstructured data processing is required to train AI models. The DL-based methods can be utilized for unstructured data sets. These strategies might limit scalability since they can be multilayered (i.e., in Multilayer Perceptron, CNN, and RNN). Besides data requirements, AI-based metamaterial design can involve highly complex design spaces to support numerous design variables, which can result in high computational costs. To address this, an approach for dimensionality reduction techniques can use a pseudoencoder, reducing computational time significantly.^277^ If these limitations are handled, automated AI-design pipelines can be fed more effectively, and their output can be utilized in a fast-track manner. These will especially improve biomedical metamaterial design for the timely intervention of patient treatment and the stealth industry, thus improving both patient care and national/international security and defense. A pilot study has shown wearable sensor usage in chronic Obstructive Pulmonary Disease (COPD) patients in in-hospital pulmonary rehabilitation programs^278^ for timely intervention and classification. Other than timely intervention, AI models can also help design more feasible and accessible materials. One example was a paper-based vertical flow assay (VFA) for high-sensitivity C-Reactive Protein (hs-CRP) testing, which is frequently used to determine the risk of cardiovascular disease (CVD), as a low-cost and quick use-case.^279^ In addition to applications in healthcare, accurate big data handling can lead to promising developments in stealth and military engineering. For instance, Wang et al. have presented a DL-assisted optimization of metamaterials for Multiband compatible infrared stealth.^280^ Reduction of the thermal signature of military targets is crucial for military engineering. In defense and combat areas, timely calibration of the thermal signature can protect military personnel.

### 6.2 Data Security and Ethical Regulations

AI-based design for biomedical applications generates and/or makes use of enormous volumes of health information through interactions with extremely sensitive patient data, which calls for safe processing, transmission, and storage.^281^ This preserves patient privacy and data security which is crucial in today’s digital environment.^282^ In addition, data safety is necessary to ensure optimal gadget operation. There are some solutions for privacy concerns such as data de-identification.^283^ The FDA and EU’s safety standards for medical devices, POC, or other regulated products must be matched for the commercialization of metamaterial designs. Especially in biomedical metamaterial design, the biased design might occur due to biased data in AI-training models and needs to be observed for regulatory compliance. Adopting cloud computing can offer flexibility for data storage and lower computational costs for on-demand scalability. On the other hand, cloud systems will cause a trade-off for power consumption and challenges for preserving the privacy of both data and the AI model. To address this, differential privacy, cryptographic techniques, and client-based federated learning techniques can be utilized for privacy-preserving DL or ML frameworks.^284,285^ As the data privacy steps increase, it might cause some complications. For example, Sabry et al. have pointed out that an ML framework, differentially private stochastic gradient descent in their case, lost certain minority classes in data. In their case, terminal patients and patients of minority ethnicities, which are usually represented in the tail of the distribution, were cut down.^281^ As such, it is essential to address bias in data and model development to avoid propagating biases. Moreover, transparency regarding the data sources, data quality, and AI algorithms used in the design process should be regulated. The EU’s General Data Protection Regulation (GDPR) may also come into play if personal data is involved in AI design.^286^ Strategies to allow model privacy,^266^ cloud security,^267^ and model reuse attacks^287^ should also be implemented.

### 6.3 Selecting Optimal AI Models

AI-based algorithms help predict the interplay between the microstructure, mechanical properties, and function of metamaterial. There is the remaining challenge of precision and accuracy since the AI-model prediction ability is directly linked to the data quality of the relation. Human interpretation is still relevant and independence is an unresolved challenge although advanced by DL^71^ and data augmentation^288^ in recent years. The understanding of these relationships with accurate prediction is important as it directly impacts design time, process, and safety. There have been some reports of frameworks with active learning-based data acquisition and tailored bias. A hybrid active-learning AI model has been reported to design high-entropy Invar alloys.^289^ The authors have emphasized the proficiency of the model with limited experimental data. The closed-loop workflow and integration ML with density-functional theory simulations, thermodynamic calculations, and experiments completed the process within months, demonstrating its proficiency in designing high-entropy Invar alloys with optimal thermal, magnetic, and electrical properties, compared to traditional methods. Another group has presented an active learning-based data acquisition framework to guide diverse data generation and property biases of the model. In the early stages of data-driven metamaterial design, data sets suffer from imbalanced property distributions. It is often that data sets are built with space-filling design and the quality of data acquisition propagates downstream. A data-driven shape descriptor was trained with generative models and a sparse regressor as a start-up agent.^290^ Furthermore, selecting the best architecture for the AI model requires experimentation and a combination of the variant parameters. One group suggested that the usage of nested-CNN with hyperparameter optimization can be promising in metamaterials. By using imputation on the prediction side, the effect of nontargeted regions was reduced but not eliminated. The aim was to develop a “no-effect” condition in the DL model. It is expected that the imputation for prediction method will provide more advantages in AI models trained in a much wider frequency range. Apart from metamaterial optimizations, such strategies can provide certain advantages for multiple-input multiple-output (MIMO) systems.^291^ However, a specific level of expertise is usually needed to establish optimal performance and desired functionality of metamaterial.

### 6.4 Transferability, Generalization, and Manufacturability

The generalization and transferability of AI models have some restrictions. It can be difficult to guarantee these models’ effectiveness and applicability across a range of metamaterial types and applications, even though they may perform satisfactorily in particular situations or data sets. If these limitations are overcome, computational sources can be cut down and utilized in further steps of the research. Reis et al. presented an inverse metamaterial design via genetic algorithms and asymptotic homogenization schemes.^54^ The variant design variables were accounted for and upon relatively low computational cost, allowed broad use. Another group suggested a Multitask DL-based design of chiral plasmonic metamaterials for generalization concerns.^213^ Another important challenge of AI-based metamaterial design is fabrication constraints. While AI-driven metamaterial designs might appear superior to conventional ones, high complexity at fabrication can limit applications. To overcome fabrication challenges, some groups utilize additive manufacturing for tunable metamaterials^292^ and Multimaterial additive manufacturing for cellular metamaterials.^293^ Other groups such as Ma et al. have utilized an inverse design framework with a deep residual network that replaces the conventional finite-element analysis for acceleration. The same DL framework was transferable for the designs of magneto-mechanical metamaterials and other active metamaterials with target mechanical, acoustic, thermal, and electromagnetic properties.^294^ Finally, cost-effectiveness, long-term reliability, maintenance requirements, and integration with existing systems should be considered in wearable metamaterial design. The bonsai tree model can be a cost-cutting approach for minimizing model size and prediction cost.^295,296^ Compression of AI models can be done by encoding and model compression techniques.^296^

### 6.5 Automatization

Challenges include the need for more high-quality data sets and computational resources required for complex AI frameworks. Due to the risk of biased data, AI models might create biased designs. The implementation of ML computation holds great promise for future design automation. It should be mentioned that, in some domains such as healthcare, fully automated frameworks, can lead to the generation of biased output. For example, Sabry et al. have pointed out that in an ML framework, differentially private stochastic gradient descent loses certain minority classes in data.^281,297^ On the other hand, in other fields of metamaterials, automation is relatively less risky than healthcare due to not interfering with the human body directly or commonly. One group reported a robotic AI-guided system for the design and manufacturing of Chiral functional film. A robotic all-round AI-Chemist could execute a full cycle process of discovering and producing chiral films. Furthermore, the film was able to achieve targeted chiroptical performance. The group suggested that the AI chemist platform can be used to produce flexible films with large chiroptical activities at a designated wavelength profile on demand.^298^ The strategy can allow various types of optical metamaterial design. The validation and interpretability of the AI output while also addressing ethical bias and regulatory compliance is essential. If not addressed early on, data or model bias can impact production and halt automatization. These bottlenecks can be improved via collaborative efforts and responsible AI practices^299^ but the gap needs to be explored further for manufacturability.

## 7 Conclusion

The development of metamaterials has achieved targeted control over electromagnetic, mechanical, and thermal properties of matter. However, metamaterial design processes have relied heavily on human intuition and manual design strategies. AI-based algorithms can accelerate the metamaterial design processes, optimize designs, and enable the development of novel metamaterial patterns. Such strategies can empower versatile applications in various fields including acoustics, optics, healthcare, and power harvesting. Selective or adaptable acoustic manipulation, increased fatigue resistance, and wider frequency ranges can be achieved in acoustics applications through the exploration of broader design spaces and dynamic metamaterial parameter manipulations. Automatic implementation of metasurfaces, lower computational costs, and nonlinear optical interactions can be accomplished using AI-based design that does not depend on time-intensive iterative strategies. The functionality, accuracy, and convenience of wearable health monitors and accurate POC sensors can also be improved if inherent structural limitations of metamaterials and fabrication bottlenecks are overcome using AI-based design. High efficiency energy harvesting or multimodal energy harvesting can be attained through the optimization of metamaterial properties. Although challenges and concerns regarding the ethical use and handling of sensitive data remain, strategies including cloud-based systems, data encryption, or international audit processes can be employed to address these issues. Similarly, while fabrication constraints and the risk for biased output generation remains, DL-based design strategies and simulations can be used to alleviate such challenges. Using optimized AI-models under ethical and legal regulations can significantly improve metamaterial functionality and facilitate applications in acoustics, optics, healthcare, and energy harvesting technologies.
